# Effectiveness of an interprofessional patient safety team-based learning simulation experience on healthcare professional trainees

**DOI:** 10.1186/s12909-018-1301-4

**Published:** 2018-08-08

**Authors:** Nirvani Goolsarran, Carine E. Hamo, Susan Lane, Stacey Frawley, Wei-Hsin Lu

**Affiliations:** 1 Department of Medicine, Stony Brook University Hospital,; 2 School of Nursing, Stony Brook University Hospital, 101 Nicolls Road, Stony Brook, 11794 NY USA; 3 Department of Preventative Medicine, Stony Brook University Hospital, 101 Nicolls Road, Stony Brook, 11794 NY USA; 4 Stony Brook University School of Medicine, 101 Nicolls Road, HSC Level 4 Room 148, Stony Brook, NY 11794-8430 USA

**Keywords:** Patient safety training, Team-based learning, Simulation-based education, Interprofessional education

## Abstract

**Background:**

Although the American Council of Graduate Medical Education (ACGME) mandates formal education in patient safety, there is a lack of standardized educational practice on how to conduct patient safety training. Traditionally, patient safety is taught utilizing instructional strategies that promote passive learning such as self-directed online learning modules or didactic lectures that result in suboptimal learning and satisfaction.

**Methods:**

During the summer of 2015, 76 trainees consisting of internal medicine interns and senior-level nursing students participated in an interactive patient safety workshop that used a flipped classroom approach integrating team based learning (TBL) and interprofessional simulated application exercises.

**Results:**

Workshop trainees demonstrated an increase in knowledge specifically related to patient safety core concepts on the Team Readiness Assurance Test (TRAT) compared to the Individual Readiness Assurance Test (IRAT) (*p* = 0.001). Completion rates on the simulation application exercises checklists were high except for a few critical action items such as hand-washing, identifying barriers to care, and making efforts to clarify code status with patient. The Readiness for Interprofessional Learning Scale (RIPLS) subscale scores for Teamwork and Collaboration and Professional Identity were higher on the post-workshop survey compared to the pre-workshop survey, however only the difference in the Positive Professional Identity subscale was statistically significant (*p* = 0.03). A majority (90%) of the trainees either agreed that the safety concepts they learned would likely improve the quality of care they provide to future patients.

**Conclusions:**

This novel approach to safety training expanded teaching outside of the classroom and integrated simulation and engagement in error reduction strategies. Next steps include direct observation of trainees in the clinical setting for team-based competency when it comes to patient safety and recognition of system errors.

## Background

Preventable medical errors are the third leading cause of death in the U.S., claiming the lives of over 400,000 patients each year [1]. These numbers underscore the need for patient safety training and education for healthcare professionals. Although the principles and concepts of patient safety are required as a critical part of healthcare education and training, many healthcare educators are uncertain how best to integrate patient safety training into their programs and curricula [2]. Patient safety training and education of healthcare professionals have not kept pace with advances in workforce requirements regarding patient safety [2].

At Stony Brook University the academic health science schools and hospital share common elements of their missions: to educate and provide excellent quality clinical care. Although there have been successful efforts to promote interprofessional learning and practice, there are still too few opportunities to systematically bring our faculty and trainees from multiple disciplines together in a meaningful way for the purpose of improving the quality of patient care by learning about each other’s roles, cultivating communication, practicing collaboration and fostering teamwork. To address this need, we designed and developed an interprofessional, collaborative, team-based learning simulation experience with the goal of promoting safe and quality patient care among healthcare professional trainees. While interprofessional simulation strategies are becoming more widely used [3–5], this training exercise demonstrates a unique way of teaching patient safety by integrating three fundamental instructional strategies: 1) team-based learning pedagogy, 2) interprofessional education (IPE) of trainees, and 3) simulation-based education (SBE).

Medicine has traditionally relied on an apprentice-style approach to learning and experience [6]. This inevitably exposes patients to inexperienced healthcare practitioners, and the dangers and harm associated with this are increasingly unacceptable [6]. Simulation is a technique to replace or amplify real-patient experiences with supervised and guided experiences, artificially contrived to evoke or replicate substantial aspects of the real world in a fully interactive manner [7]. Furthermore, interprofessional communication and collaboration is crucial to patient safety training, especially when they enter clinical practice [8]. According to the Interprofessional Education Collaborative (IPEC) [9], the ability to work with other health professionals to maintain a climate of mutual respect and shared values; understand one’s own role and those of other professions in order to appropriately address the healthcare needs of the patients; use a team approach to communicate with patients and their families; and perform a variety of team roles to deliver patient-centered care are the four core competencies for inteprofessional collaborative practice. Training future healthcare providers to work in interdisciplinary teams that help enhance these competencies should facilitate improved healthcare outcomes for patients [3]. Hence, interprofessional education coupled with simulation application allows for the collaboration of expertise from different fields and the demonstration of these core competencies in safety education.

Team-based learning (TBL) is an evidence-based, multiphase pedagogical approach that requires active learner/trainee participation and collaboration [4]. This instructional strategy is an ideal way to promote team science and collaborative efforts aimed to address a scientific challenge that leverages the strengths and expertise of professionals trained in different fields [4]. TBL is a flipped classroom model that utilizes a readiness assurance process intended to hold learners accountable for coming to class prepared. Prior to class, the learners are provided materials to study based on the learning objectives of the class. The class begins with a readiness assurance process that consists of an Individual Readiness Assurance Test (IRAT) given to individual learners to assess key concepts that they are expected to acquire from the pre-class study materials. Immediately after the IRAT, the learners take the same test in teams (Team Readiness Assurance Test) by discussing amongst themselves and coming to a consensus on the answer to each question. After the readiness assurance process, the teams then work on application-based exercises that promote higher level learning and student engagement.

The objective of this study was to design, implement, and evaluate the effectiveness of a simulation training model that incorporates interprofessional learning and TBL for interdisciplinary faculty to teach core concepts of patient safety.

## Methods

**Fig. 1:**
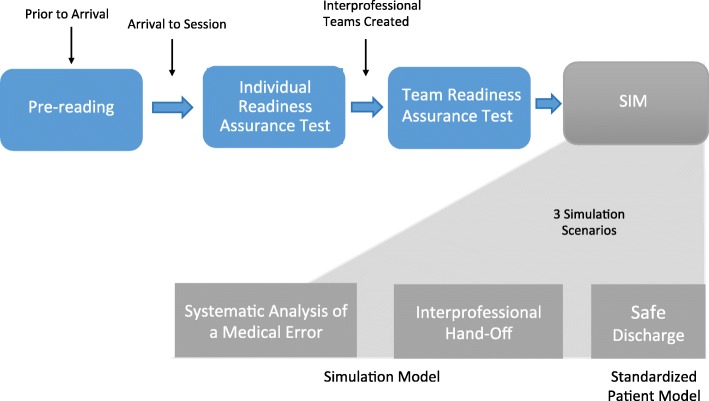
Study flow of the project design and implementation

During the months of July and August 2015, we conducted five 3.5-h interprofessional patient safety-training workshops. These workshops were scheduled during residents’ protected didactic time. At each workshop, trainees (incoming internal medicine interns and senior-level nursing students) were randomly grouped into pre-determined interprofessional teams consisting of 4–6 trainees per team. The size of each team varied depending on the number of trainees available for the workshop. Each team had either one or two internal medicine residents and between three or four nursing students. Prior to this workshop the trainees had not worked together before and were not given the team assignments in advance. The teams remained together for the duration of the workshop, rotating through all of the clinical simulation scenarios as a unit (Fig. 1). By the end of the workshop, trainees were expected to be able to demonstrate the following learning objectives: 1) develop a root cause analysis and generate an action plan, 2) conduct a patient hand-off as the transmitter and receiver, and 3) demonstrate patient teach-back and safe discharge instructions [10].

### Sample

Our study participants consisted of a convenient sample of 26 first year internal medicine residents and 50 undergraduate nursing students enrolled in an upper-division, accelerated one-year baccalaureate nursing program. According to the Joint Commission, communication failures have been identified as the top contributing factor to sentinel events reported [11]. Therefore these two groups were chosen based on a call from the Joint Commission asking for increased collaborative training in physician-nurse communication. Within one year post-study, the nursing student participants will be eligible to sit for State licensure and subsequently be employed as Registered Nurses, where the need to communicate using succinct and effective language is critical and will directly impact on patient safety. The internal medicine residents have just started their intern year and therefore such training is essential for safe clinical practice. Prior to this workshop, these participants did not have any formal patient safety education.

### Intervention and data collection

The pre-workshop study materials included readings of evidence-based literature and voice over powerpoints on topics regarding effective handoffs, safe discharge and systemic analyses of medical errors. In adherence with team-based learning pedagogy, an Individual Readiness Assurance Test (IRAT) was completed by each trainee, followed by an identical Team Readiness Assurance Test (TRAT) that was completed by each interprofessional team. The IRAT/TRAT consists of 10 questions, each question worth 1 point for a total possible score of 10 points. The content of the test is based on patient safety literature contained in pre-reading assignments given to the trainees one week prior to the workshop. A content expert reviewed the IRAT/TRAT for face and content validity. At the end of the TRAT, faculty instructors reviewed the correct and incorrect answers and provided feedback, rationale, and offered clarifications to the trainees. The purpose of the IRAT/TRAT was to hold the trainees accountable for learning the pre-reading materials and to demonstrate understanding of the concepts with their team members in preparation for the simulation case scenarios.

Following the readiness assurance process, however, rather than a TBL application-based exercise on paper, the interprofessional teams engaged in three simulations using high fidelity manikins and standardized patients designed to reinforce the workshop learning objectives through hands-on teamwork and communication. These simulation cases included: 1) systematic analysis of a medical error with the use of a high fidelity mannequin, 2) interprofessional I-PASS mnemonic handoff [12] with the use of a high fidelity mannequin, and 3) safe discharge using patient teach-back techniques with the use of a standardized patient (Table 1). Performance checklists were developed for each simulation case based on the learning objectives completed in the pre-reading materials, as well as components of TeamSTEPPS [12]. After each simulation case the trainees participated in immediate post-scenario debriefing with interprofessional faculty instructors using a critical action checklist developed for each scenario. This provided the interprofessional team members with the opportunity to reflect upon and discuss their experiences and consider what they might do differently in the future. It also gave faculty instructors to the opportunity to comment on certain interactions they observed such as communication and interpersonal skills.

**Table 1:** Brief narrative description of the Simulation/Standardized Patient Application Cases

Case1: Systematic Analysis of a Medical Error (requires high fidelity simulation mannequin)	
Overall Goal: Understand the impact of a medical error, conduct a root cause analysis and generate an action plan to prevent future errors. Scenario: A 45-year-old male who was admitted for shortness of breath. He was found to have a right main stem pulmonary artery embolism. He has been on intravenous unfractionated heparin for the past 48 h without proper dose adjustment. His aPTT has been < 20 s since admission. The heparin dose was incorrectly calculated when initially ordered by the intern. In addition, nursing staff and pharmacy subsequently failed to adjust the dose of heparin for 48 h. He is now in Pulseless Electrical Activity (PEA) arrest. Your team is being called into a code blue/cardiac arrest. Your task is to follow ACLS protocol with your team. Following this, you will conduct a root cause analysis of the event. Your main task is to identify possible contributing causes of the error and create a root cause analysis (RCA) using the fishbone diagram provided. Following discussion of the RCA, the group will suggest an action plan to prevent this error from occurring in the future.	
Case 2: Interprofessional Hand-off (requires high fidelity simulation mannequin with voice control)	
Overall Goal: Provide written and verbal hand-off using a standardized format. Scenario**:** A 76-year-old male with past medical history of severe COPD, CAD s/p CABG, Diabetes type 2, peripheral artery disease, and hypertension, who was admitted early this morning for acute on chronic respiratory failure due to progression of his underlying severe COPD. Patient was placed on supplemental oxygen via venti-mask, albuterol/ipratropium nebulizers every 4 h and given IV solumedrol (methylprednisolone). He appears to be stable for now, but is very sleepy and has been deferring all questions to his wife. Your task is to review the list of interval events throughout the day and generate an IPASS hand-off for the incoming team using the IPASS format.(12)	
Case 3: Safe Discharge (requires a standardized patient actor)	
Overall Goal: Demonstrate patient teach back and safe discharge planning Scenario: A 56-year-old white male is newly diagnosed with type 2 diabetes. At 200 lbs., he has a history of hypertension and hyperlipidemia. He was admitted 2 days ago for uncontrolled blood glucose levels requiring IV insulin. His AIC is 11% and has not responded to PO diabetic medications in the past. He is now ready to be discharged. The nurse provided teaching on how to manage diabetes at home. She has given him instructional materials, including the brochures “Facts about Diabetes” and “How to manage diabetes at home.” The patient has also watched videos that cover basic information about diabetes management, such as how to use the blood glucose meter. Patient is ready to be discharged but he feels very overwhelmed by the information provided to him. You are the day team on the general medicine floor. Your task is to make sure he is all set to go home and answer any questions he may have.	

### Curriculum evaluation

The simulation training program was evaluated using data collected from trainees on their knowledge, skills, and attitudes. Specifically, patient safety knowledge-based learning outcomes were evaluated using the IRAT and TRAT. The rationale for comparing IRAT and TRAT was to evaluate the contribution to learning from the interprofessional team experience and to provide corrective feedback to the team.

In terms of attitudinal data, the Readiness for Interprofessional Learning Scale (RIPLS), a validated 19-item questionnaire developed by Parsell and Bligh [13, 14], was administered immediately before and after the workshop. RIPLS uses a 5-point Likert scale to provide healthcare professional students the opportunity to self-rate from Strongly Agree (5) to Strongly Disagree (1) on statements regarding their knowledge, skills, and attitudes on working with other healthcare professionals. McFayden et al. [15] adopted the original version of the questionnaire to reflect four subscales: (a) Teamwork and Collaboration (9 items) that measures the knowledge and skills needed to participate in interprofessional learning, (b) Negative (3 items) and Positive Professional Identity (4 items) that measures perception of the values and benefits of interprofessional learning, and (c) Roles and Responsibilities (3 items) that measures perceptions of what can be performed in an interprofessional learning environment. Note that for Negative Professional Identity subscale, two statement items are negatively worded and therefore lower ratings reflect more favorable attitudes.

The trainees completed a post-workshop self-assessment survey that we developed that asked them to rate on a scale of 1 (Not Confident at All) to 5 (Very Confident) their confidence level in carrying out the concepts learned during the simulation workshops. Finally, completion rates of the performance checklist for each simulation case allowed trainees to apply and demonstrate hands-on skills of what they have learned from the pre-workshop study materials and then reinforced through the readiness assurance process.

### Statistical analysis

Data were analyzed using IBM SPSS Statistics for Windows, Version 22.0 (Armonk, NY: IBM Corp). Descriptive statistics were used to calculate mean scores, standard deviations and percentages. As individual identifiers were not collected on the readiness assurance tests, an independent samples *t*-test was used to compare the overall mean IRAT scores with the overall mean TRAT scores to see if there was a difference between individual and team performances. Chi-square tests were conducted to compare the number of correct responses for each question item between the IRAT and TRAT. Aggregate pre-post responses for the Readiness for Interprofessional Learning Scale (RIPLS) [13] were analyzed using an independent samples *t*-test. A significant level of *p* < 0.05 was considered to be statistically significant.

## Results

A convenience sample of a total of 76 trainees (26 medicine interns and 50 senior-level nursing students) participated in the workshops, creating twenty interprofessional teams. All analyses were calculated based on data collected from the 76 workshop trainees.

### IRAT/TRAT scores

The TRAT scores (Mean = 7.7, SD = 1.8) were significantly higher than the IRAT scores [Mean = 5.6, SD = 1.7; *t*(94) = − 4.9, *p* = .001]. A significantly higher number of correct responses were selected on the TRAT compared to the IRAT on questions related to these patient safety concepts: preventable adverse events, cognitive biases, and patient handoff communication [5].

### Critical action checklists

Performance of each interprofessional team on the three simulation/standardized patient application case scenarios was assessed through direct observation by MD and RN faculty instructors using checklists that included critical action items. After each interprofessional team performance, the faculty instructors would immediately discuss what they observed and come to a 100% full consensus on which items were completed by the team. A majority of the critical action items on the checklists were completed by the interprofessional teams (see Table 2).

**Table 2:** Completion of Application Case Checklist Action Items by Interprofessional Teams (*n* = 20)

	Actions Completed (%)
Critical Action Items: Systematic Analysis of a Medical Error	
Assessed initial vital signs	95%
Secured airway	95%
Appropriate ACLS protocol	80%
Asked for patient history	55%
Wash hands	45%
Obtain focused PE	95%
Recognize medical error/adverse event	100%
Identify contributors of the medical error	100%
Identify strategies to decrease probability of this error	100%
Generate a root cause analysis	100%
Critical Action Items: Interprofessional Hand-Off	
Provided appropriate illness severity	55%
Patient summary is concise and relevant	55%
Provided action plan with clear instructions	100%
To do list provided	100%
Provided situation awareness – plan for what might happen	100%
Made efforts to clarify code status with patient (does not align with IPASS)	40%
Allowed receiver to synthesize and summary	95%
Allowed receiver to ask questions	100%
Introduced himself/herself and explained roles	80%
Critical Action Items: Safe Discharge	
Include patient as a full partner in the discharge process	95%
Identified barriers to care	50%
Reviewed medication regimen	95%
Highlight warning signs and problems (hypoglycemia or hyperglycemia symptoms)	95%
Discuss follow up appointments	95%
Educate the patient in plain language about condition (no medical jargon used)	100%
Assess patient understanding/patient teach back (asked patient to repeat back)	70%
Provide information in small chunks and repeat key pieces of information	95%

### RIPLS scores

Although the RIPLS scores for the Teamwork and Collaboration and Professional Identity subscales (Negative and Positive combined) were higher on the post-workshop survey (Mean = 42.9, SD = 3.5; Mean = 22.8, SD = 2.7 subsequently) compared to the pre-workshop survey (Mean = 41.9, SD = 4.4; Mean = 22.0, SD = 2.6 subsequently), a statistical difference was seen only in the Positive Professional Identity subscale from before (Mean = 17.4, SD = 2.2) to after the workshop (Mean = 18.3, SD = 2.2; *t* (140) = − 2.2, *p* = 0.03) (Table 3).

**Table 3:** The Readiness for Interprofessional Learning Scale (RIPLS) Pre and Post Workshop Survey Scores

	Teamwork and Collaboration subscale		Negative Professional Identity subscale		Positive Professional Identity subscale		Roles and Responsibilities subscale		RIPLS Total Score	
	Pre	Post	Pre	Post	Pre	Post	Pre	Post	Pre	Post
All (*n* = 76)	41.9 (4.4)	42.9 (3.5)	4.6 (2.0)	4.5 (2.2)	17.4 (2.2)	18.3* (2.2)	9.8 (1.9)	9.8 (2.1)	73.8 (5.8)	75.5 (5.9)
Medicine Interns (*n* = 26)	41.9 (3.2)	42.7 (3.3)	4.9 (2.3)	5.2 (2.6)	17.5 (2.3)	18.3 (2.0)	10.1 (2.0)	10.7 (2.4)	74.4 (5.8)	76.9 (6.0)
Nursing students (*n* = 50)	41.9 (4.9)	43.0 (3.6)	4.5 (1.9)	4.1 (1.8)	17.4 (2.2)	18.3 (2.3)	9.6 (1.8)	9.3 (1.8)	73.5 (5.9)	74.8 (5.8)

*indicates p < 0.05

Note: Original subscale scores are presented to better reflect the nature of each construct

### Self-assessment

In the post-workshop self-assessment survey, over 90% of the participants either ‘agreed’ or ‘strongly agreed’ to the statement “The safety concepts I learned will impact my clinical practice”. Additionally, a majority of the workshop participants agreed that they felt confident in “conducting error analysis and root cause analysis” (78%), “their ability to provide proper hand-off using IPASS” (86%), and “their ability to provide a safe discharge plan” (86%).

## Discussion

Learning about patient safety requires the understanding of relevant concepts and principles as well as the demonstration of teamwork, communication skills, and error reduction strategies. This study used simulation-based education that integrated two instructional strategies to teach patient safety: team-based learning and interprofessional, collaborative learning. Additionally, the simulation application is centered on commonly encountered medical errors. Simulation is a powerful and frequently used technique that can help healthcare professionals achieve higher levels of competence and safer care [2]. Our study incorporates structured team-based pedagogy with interprofessional learning in order to emphasize realistic team communication, decision-making, judgment, and leadership skills necessary to handle medical errors [4].

Team performance on the TRAT for patient safety knowledge was higher than that of the individual performance on the IRAT. The team-based learning approach was used to ensure that trainees would begin the workshop with a basic understanding of patient safety concepts including systematic analyses of medical errors, effective handoffs, and patient education teach-back techniques. The simulation/standardized patient application case scenarios that followed required the trainees to access and apply patient safety, communication, and teamwork skills. One of the benefits of the TBL approach ensures that misunderstanding and/or lack of understanding of certain concepts at the individual level, is addressed in discussion and peer learning during the team readiness assurance process. Previous research also showed that learners perform better on the TRAT if they complete the IRAT first because learners would need to consider the questions on their own first and therefore feel inclined to contribute to the team discussion to share their thoughts as well as listen to the thoughts of others [16]. This process facilitates acquisition of common and complete understanding of the important concepts among team members as they move forward with the application exercises. The TBL approach also encourages interprofessional communication and learning to occur; allowing different healthcare professionals the opportunity to bring their discipline-specific perspective to the team discussions so that as a team everyone is in agreement about how to best coordinate and provide patient care. Higher performance on the TRAT was to be expected in our study and supports findings from the literature that TBL is an effective strategy to promote interprofessional collaboration [4].

We found high completion rates for a majority of the critical safety actions across all interprofessional teams for each case scenario. There were a few action items that were not performed by at least half of the interprofessional teams such as hand washing (45% of interprofessional teams completed), clarifying code status with the patient (40% of interprofessional teams completed); and identifying barriers of care with the patient (50% of interprofessional teams completed). Additionally, only 55% of the interprofessional teams were able to provide appropriate illness severity and give a patient summary that is concise and relevant. This information can inform health professions educators as they design and develop training programs and curriculum. Hand washing, in particular, is a simple, yet critical, action item that should be continuously emphasized in all areas of simulated training programs, including patient safety curricula.

While increases in the RIPLS Teamwork and Collaboration and Professional Identity subscales were noted after workshop participation, the only significant difference occurred in the Positive Professional Identity subscale. According to the definition of each subscale and description of its psychometrics properties provided by Parsell and Bligh [13], these results suggest that after participating in the workshop, trainees’ perceptions of shared learning with other healthcare professionals positively increased, viewing interprofessional learning as useful for improving communication and problem solving skills [13]. Additionally, although the Roles and Responsibilities subscale overall scores did not change (Mean = 9.8), the subscale score increased for the medicine interns (from 10.1 to 10.7) and decreased for the nursing students (from 9.6 to 9.3) after the workshop compared with before. Lower scores on the Roles and Responsibilities subscale imply a clearer perception of one’s role and those of others [13]. While McFadyen [15] suggests that the internal consistency for this subscale is weak if it is used to examine undergraduate students (due to a lack of a clear understanding of what their roles and responsibilities are), it is interesting that the medicine interns, who are post graduate level trainees, were less clear of their role and responsibilities compared to the senior level nursing students. This may be because the interns were just beginning their training compared to the nursing students who were starting their senior year of training. Participation in the interprofessional simulation exercises may have reinforced perceptions of one’s contributions to the team, or reinforced the concept that delivery of safe patient care is the responsibility of the team as a whole, and not by individuals working independently. Trainees reported a very positive experience with the workshop and indicated that the patient safety content covered will likely impact the care they provide to patients in the future.

This study has several limitations that include a small sample size from each profession at a single institution and the use of a pre-posttest study design which does not assess the long-term effect of the intervention. Some caution is therefore advised in interpreting the results of this study as these results may not be generalizable to the general population in each profession. Some existing literature also questions the use of the RIPLS instrument and its sensitivity to detect meaningful change over time since researchers frequently find no significant difference between pre-and post IPE intervention [17]. In our study, however, we did see statistically significant improvements for the Positive Professional Identity subscale after a brief 3.5 h workshop. Last, limitations to implementing this approach to patient safety training include accessibility of a simulation environment, standardized patients, and faculty availability in which replication at other institutions that do not have such resources may be challenging. Furthermore, the trainees’ ability to apply key principles of patient safety in actual clinical settings was not assessed in this study.

Next steps include repeat interprofessional, TBL simulations throughout the course of training, follow-up with trainees to obtain their perspectives on whether this activity has impacted patient care, and direct observation of residents in actual patient care settings including discharge and hand-off experiences. In this way, trainees can be further observed in actual clinical settings for team-based competency in patient safety and recognition of systems errors.

Future directions should include the development of patient safety direct observation tools to evaluate whether trainees are applying the learned simulation skills in their clinical practice. These tools can be used to measure skills during routine patient care on clinical units. Patient care events including error disclosure, multidisciplinary meetings, patient discharge meetings, and patient counseling about safety issues may be observed and evaluated.

## Conclusions

Through interprofessional, TBL simulation, trainees actively participate, engage in, and demonstrate learning concepts related to patient safety. Implementation of such an approach is an important teaching strategy which can increase healthcare trainees’ interest and motivation to learn about key patient safety concepts. This training program will fulfill educational requirements and promote necessary skills among trainees to develop lifelong habits to effectively reduce medical errors. In this study we found that a simulation model in an interprofessional team setting can teach healthcare professionals realistic, hands-on principles of patient safety.

## Abbreviations

IPE: Interprofessional education
IRAT: Individual Readiness Assurance Test
RIPLS: Readiness for Interprofessional Learning Scale
SBE: Simulation-based Education
TBL: Team-based Learning
TRAT: Team Readiness Assurance Test
